# Ganglioside GD2 expression is maintained upon recurrence in patients with osteosarcoma

**DOI:** 10.1186/s13569-014-0020-9

**Published:** 2015-01-24

**Authors:** Vincent I Poon, Michael Roth, Sajida Piperdi, David Geller, Jonathan Gill, Erin R Rudzinski, Douglas S Hawkins, Richard Gorlick

**Affiliations:** Division of Pediatric Hematology/Oncology, Children’s Hospital at Montefiore, Albert Einstein College of Medicine, 3415 Bainbridge Ave, Rosenthal Pavilion, Room 300, Bronx, NY 10467 USA; Department of Orthopaedic Surgery, Montefiore Medical Center and the Children’s Hospital at Montefiore, Albert Einstein College of Medicine, Bronx, NY USA; Department of Pathology, Seattle Children’s Hospital and University of Washington, Seattle, WA USA; Division of Pediatric Hematology/Oncology, Seattle Children’s Hospital, Fred Hutchinson Cancer Research Center, and University of Washington, Seattle, WA USA; Department of Pediatrics and Molecular Pharmacology, Albert Einstein College of Medicine, Bronx, NY USA

**Keywords:** Osteosarcoma, Ganglioside GD2, Immunotherapy, Antibody

## Abstract

**Background:**

Osteosarcoma is the most common primary malignant bone tumor in children and young adults. Ganglioside GD2 has been previously found on the cell surface in various tumor types, including osteosarcomas.

**Findings:**

In this study, forty-nine additional osteosarcoma samples from 14 individual patients were assessed for GD2 expression via immunohistochemistry, of which 47 samples were found to express GD2. In matched samples from patients, GD2 expression seen at initial biopsy was found to persist in 100% of tissues taken at recurrence.

**Conclusions:**

GD2 expression was found to persist upon recurrence. These results suggest a phase 2 trial in children with recurrent osteosarcoma should provide an appropriate read out on the efficacy of anti-GD2 antibody.

**Electronic supplementary material:**

The online version of this article (doi:10.1186/s13569-014-0020-9) contains supplementary material, which is available to authorized users.

## Findings

Osteosarcoma is the most common primary malignancy of the bone in children and young adults. Survival outcomes for children with osteosarcoma have remained stagnant for the past three decades. Novel therapeutic approaches are needed to improve outcomes for these patients. Studies have demonstrated that targeting the ganglioside GD2 in patients with high risk neuroblastoma improves survival [1]. Our group recently reported that GD2 is expressed in osteosarcoma, and the data suggested GD2 expression may be increased in relapsed specimens. However, the previous study lacked matched samples to make definitive conclusions [2].

One challenge in molecularly targeted therapy is the “moving target” of the changing proteome of cancer cells. Whether they stem from further acquisition of mutations, genetic instability, microenvironmental factors, epigenetic changes, or immunoediting, changes in cell surface protein expression of recurrent tumors may lead to resistance against targeted therapies [3-6]. In this study, we compared matched primary tumor samples to recurrent tumors to elucidate the stability of GD2 expression.

## Materials and methods

### Patient samples

An osteosarcoma tissue microarray (TMA) was constructed at Seattle Children’s Hospital, Seattle, WA with prospective approval from the local Institutional Review Board. The TMA was constructed to include tumor tissue obtained at the time of initial diagnosis (from either the primary or metastatic site), at the time of definitive surgery, and at the time of disease recurrence (either local or metastatic). In total 119 cores from 57 separate specimens (of which 49 specimens were deemed useable following the construction and staining process) representing 14 different patients were included in construction of the TMA, as shown in Additional file 1: Figure S1. Each core was included as a separate data point in the analysis.

The TMAs were constructed using 2 mm cores obtained from the original formalin-fixed paraffin-embedded tissue block for each individual specimen. Each specimen was sampled in at least duplicate. Four micron thick unstained sections were then cut from the TMA block for subsequent immunohistochemical studies.

### Immunohistochemistry

The tissue microarray slides were stained for GD2 expression using 100ug/ml of 14.G2a antibody as previously described [2]. 14.G2a antibody was a generous gift from Dr. Karen Muszynski at the National Cancer Institute. Scoring was performed using a Nikon Inverted Microscope ECLIPSE TE200 (Nikon Instruments Inc, Tokyo, Japan) attached to a CCD (Diagnostic Instruments, Sterling Heights, Michigan) by three scientists with experience scoring tissue microarrays.

Tissue samples were scored as negative (−) if there was no staining seen. A melanoma section known to express GD2 was used as a positive control and reference for positive staining. Samples were scored as positive (+++) if 67% to 100% of the section demonstrated the same intensity and distribution of staining. Slides were considered intermediate (++) if 34% to 66% of cells stained positive, and were scored as sporadic (+) when only 1% to 33% of cells stained positive. Tissue sections were graded by 3 independent observers blinded to the patient information. Clinical data for correlative analyses were obtained from Seattle Children’s Hospital only after all grading had been completed.

### Statistical analysis

Immunohistochemical assays for GD2 were assessed by three independent observers, and the level of variability between individuals was assessed using a two-factor ANOVA without replacement. When there was disagreement between the 3 observers, the median value was selected as the final tissue grading. Duplicate sample cores were assessed for agreement, and in cases of discordance, the higher value was selected as the final grading. A Mann–Whitney U-test was used to determine differences in GD2 expression levels in the primary biopsy specimens compared with the recurrent disease specimens. A *P* value of < 0.05 was considered to be statistically significant.

## Results

### Demographics

Forty-nine samples from 14 osteosarcoma patients re-mained for examination, taken at primary biopsy (10% of samples), metastases at diagnosis (4%), primary disease treated with neoadjuvant therapy at the time of definitive surgery (20%) and at disease recurrence (65%) (Table 1). Five patients with samples from initial biopsies with matched recurrent samples were available for evaluation in the current study; two of recurrent sample cores were from local recurrences (16%), and the remaining sample cores were from distant lung metastases (84%). One patient, with 2 samples from different regions of the tumor in the initial biopsy, did not have a biopsy from disease recurrence available for comparison.

**Table 1:** **GD2 expression in osteosarcoma samples assessed by immunohistochemistry**

	**Unique cores**	**-**	**+**	**++**	**+++**
**Primary**	5	0	1	4	0
**Metastases at Diagnosis**	2	0	1	0	1
**Treated resection**	10	1	3	3	3
**Recurrent**	32	1	7	12	12

Mean patient age was 14.2 years (range 7–19) and 71% of patients were male. Tumor histology was classified as osteoblastic (64%) or chondroblastic (36%) and the most common primary tumor sites were the femur (64%), tibia (21%), humerus (7%) and pelvis (7%). All patients were treated with high-dose methotrexate, doxorubicin and cisplatin, with one patient receiving additional ifosfamide, and two patients receiving additional ifosfamide and etoposide. Additional patient characteristics can be seen in Additional file 2: Table S1.

### GD2 expression

**Figure 1:**
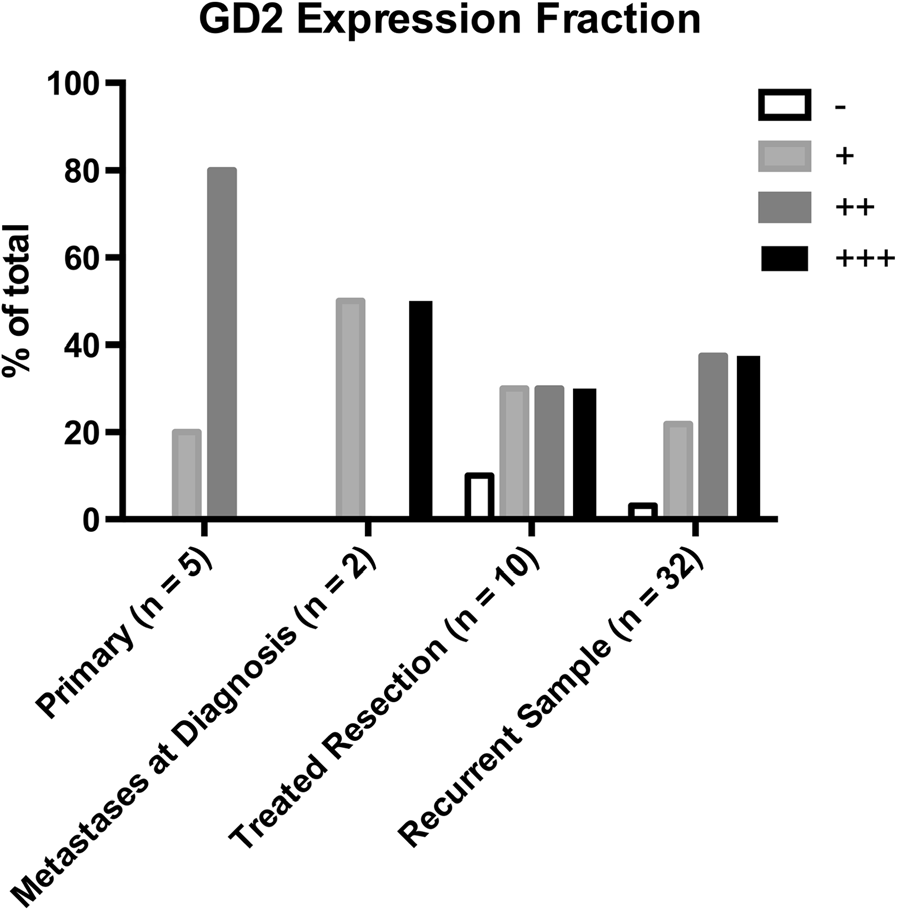
**Expression of GD-2 in osteosarcoma cores.** Cores taken from the primary biopsy, metastases at diagnosis, treated resection and upon recurrence were stained with a GD-2 specific antibody and examined via immunohistochemistry. Three independent observers scored the samples on a scale from – to +++. No significant difference in expression was seen between primary biopsy/treated resection samples versus recurrent samples (p = 0.15).

**Figure 2:**
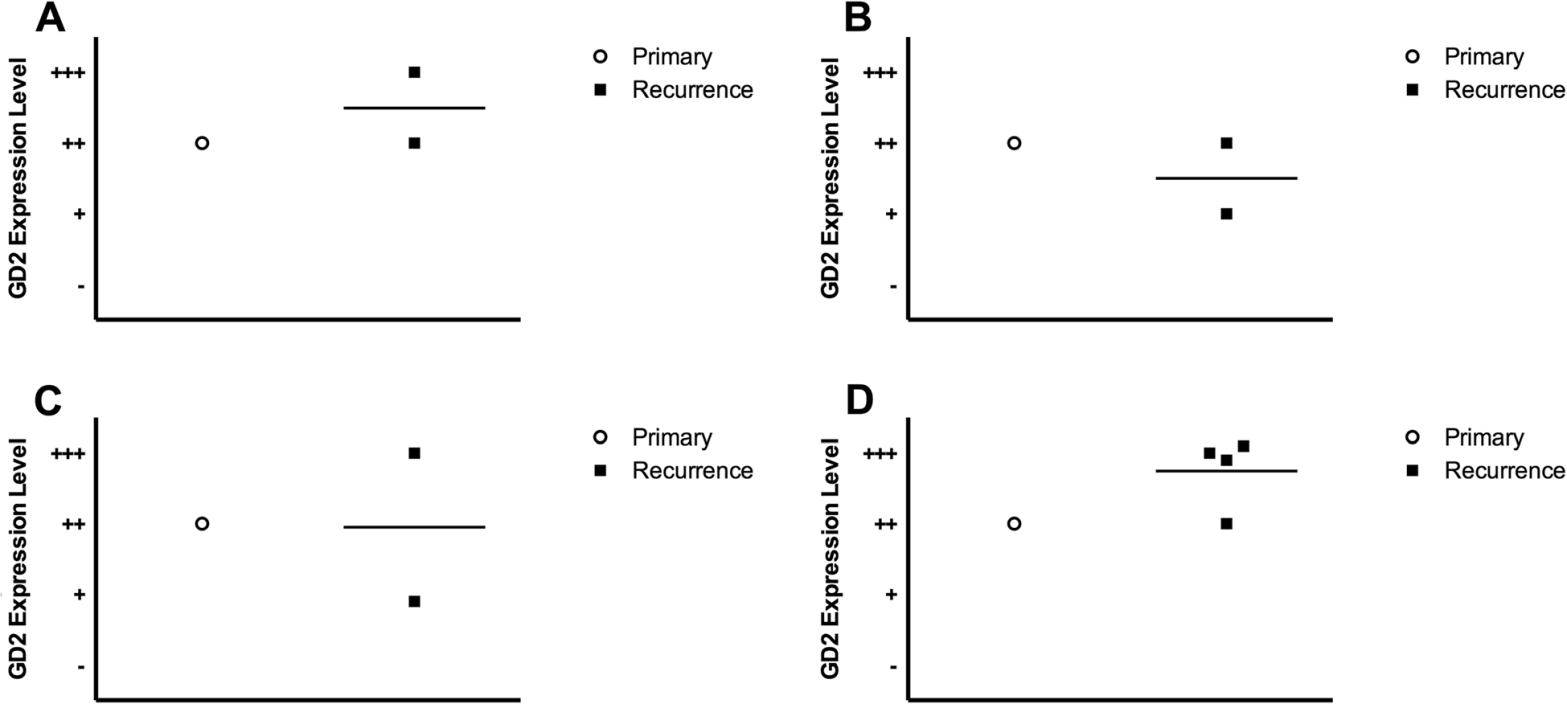
**Variation in GD-2 expression between primary and recurrent tumor cores from the 4 patients with matched samples.** Each data point represents one unique core, taken either from the primary biopsy or from a single recurrent sample. Panels **A-D** indicate unique patients. The recurrent samples shown in panel **A** were taken from local recurrence, while panels **B-D** show patients with distant lung metastases.

The level of variability between three independent observers was assessed to be non-significant using a two-factor ANOVA without replacement (p = 0.24), and the intraclass correlation coefficient was found to be 0.72, suggesting a fair to good level of agreement. The tissue microarray of 49 samples stained with the monoclonal antibody 14G2A demonstrated GD2 expression in 95% of samples. Ninety-seven percent of all recurrent disease specimens analyzed expressed GD2, however, the level of expression was not significantly different (p = 0.15) between initial biopsy samples compared with treated resection samples (Figure 1). Recurrent disease specimens demonstrated varied expression of GD2 amongst core biopsies from the same patient. Level of GD2 expression was not significantly different between initial primary biopsy specimens and matched recurrent disease specimens, whether the recurrence was local (Figure 2A) or distant (Figure 2B-D).

## Discussion

Over the past few decades there has been limited improvement in outcomes for patients with osteosarcoma. The identification of specific molecular targets has the potential to improve patient outcomes with the use of novel treatment strategies. The current data demonstrate that the surface protein ganglioside GD2 is stably expressed in osteosarcoma [2]. This provides a rationale for assessing the efficacy of anti-GD2 antibody therapy in osteosarcoma patients with recurrent disease.

In contrast to the prior report, samples did not show increased levels of GD2 expression upon recurrence. Matched cores from recurrent samples showed varying expression of GD2, with no significant change of expression compared to cores from the initial biopsy. The variability in expression in the cores taken at recurrence may be due to the intratumoral heterogeneity, or variability in the percentage of tumor versus stroma included in the cores, as the location of the core relative to the tumor architecture may have been reflected in variation in the local tumor microenvironment. Future studies could utilize multicolor IHC in order to identify possible intratumoral factors that influence GD2 expression. It is possible that GD2 expressing cells represent a subset of osteosarcoma cells or areas of highly proliferative cells [7,8]. In breast cancer, a number of groups have suggested that GD2 may be a marker of cancer stem cells, making them an especially attractive therapeutic target [9,10]. In such cases, examining markers for proliferation, such as Ki-67, or cancer stem cell markers, such as CD44, may elucidate potential intratumoral effects.

As therapies targeting GD2, such as the therapeutic antibody CH14.18, begin entering clinical testing in patients with osteosarcoma, monitoring potential loss of GD2 expression on osteosarcoma cells may aid in predicting possible development of treatment resistance. Interestingly, in patients with neuroblastoma, GD2 expression was maintained even after treatment with the anti-GD2 antibody 3 F8 [11].

The current study is limited by overall sample numbers, and it is possible that our analyses lacked the power to delineate differences in GD2 expression. However, coupled with our previous studies, it is clear that GD2 is expressed in both primary and recurrent osteosarcoma [2].

Given the near universal expression of GD2 in both primary and recurrent osteosarcoma samples, it is unlikely that GD2 expression is a prognostic biomarker, but the relatively limited number of samples examined makes this difficult to assess. However, the expression of GD2 on nearly all primary and recurrent osteosarcoma specimens suggests it is an attractive target for antibody-mediated therapy. Clinical trials are needed to assess the efficacy of anti-GD2 antibody therapy in patients with osteosarcoma.

## Additional files

Additional file 1: Figure S1. Workflow of the tumor biopsy sectioning, scoring, and analysis. Additional file 2: Table S1. Clinical characteristics of the patient cohort.

## Abbreviation

TMA: Tissue microarray
