# Supplementary figures and images for: Ganglioside GD2 expression is maintained upon recurrence in patients with osteosarcoma
Source: Clin Sarcoma Res. 2015 Jan 24;5:4. doi: 10.1186/s13569-014-0020-9 (PMC4311500; doi:10.1186/s13569-014-0020-9)

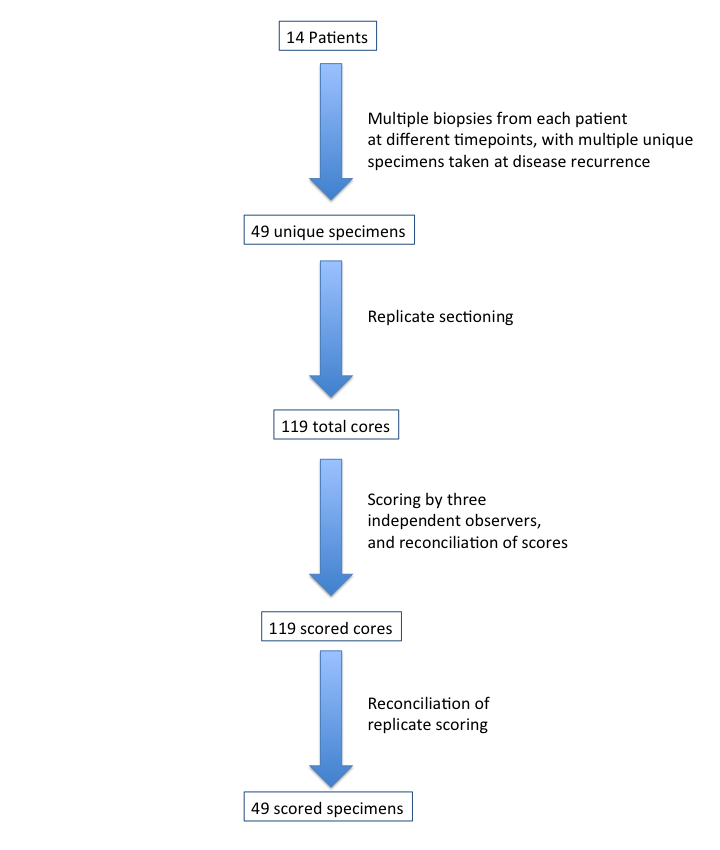

Supplement: Additional file 1: Figure S1. — Workflow of the tumor biopsy sectioning, scoring, and analysis. [file 13569_2014_20_MOESM1_ESM.png]
